# Single-strand specific nuclease enhances accuracy of error-corrected sequencing and improves rare mutation-detection sensitivity

**DOI:** 10.1007/s00204-021-03185-y

**Published:** 2021-11-12

**Authors:** Yuki Otsubo, Shoji Matsumura, Naohiro Ikeda, Masayuki Yamane

**Affiliations:** 1 R&D Safety Science Research, Kao Corporation, 3-25-14 Tono-machi, Kawasaki-ku, Kawasaki City, Kanagawa 210-0821 Japan; 2 R&D Safety Science Research, Kao Corporation, 2606 Akabane, Ichikai-Machi, Haga-Gun, Tochigi, 321-3497 Japan

**Keywords:** Next-generation sequencing, Rare mutation, Error-corrected sequencing, Single-strand specific nuclease, End-repair artifacts, Mutagenesis

## Abstract

Error-corrected sequences (ECSs) that utilize double-stranded DNA sequences are useful in detecting mutagen-induced mutations. However, relatively higher frequencies of G:C > T:A (1 × 10^−7^ bp) and G:C > C:G (2 × 10^−7^ bp) errors decrease the accuracy of detection of rare G:C mutations (approximately 10^−7^ bp). Oxidized guanines in single-strand (SS) overhangs generated after shearing could serve as the source of these errors. To remove these errors, we first computationally discarded up to 20 read bases corresponding to the ends of the DNA fragments. Error frequencies decreased proportionately with trimming length; however, the results indicated that they were not sufficiently removed. To efficiently remove SS overhangs, we evaluated three mechanistically distinct SS-specific nucleases (S1 Nuclease, mung bean nuclease, and RecJf exonuclease) and found that they were more efficient than computational trimming. Consequently, we established Jade-Seq™, an ECS protocol with S1 Nuclease treatment, which reduced G:C > T:A and G:C > C:G errors to 0.50 × 10^−7^ bp and 0.12 × 10^−7^ bp, respectively. This was probably because S1 Nuclease removed SS regions, such as gaps and nicks, depending on its wide substrate specificity. Subsequently, we evaluated the mutation-detection sensitivity of Jade-Seq™ using DNA samples from TA100 cells exposed to 3-methylcholanthrene and 7,12-dimethylbenz[a]anthracene, which contained the rare G:C > T:A mutation (i.e., 2 × 10^−7^ bp). Fold changes of G:C > T:A compared to the vehicle control were 1.2- and 1.3-times higher than those of samples without S1 Nuclease treatment, respectively. These findings indicate the potential of Jade-Seq™ for detecting rare mutations and determining the mutagenicity of environmental mutagens.

**Supplementary Information:**

The online version contains supplementary material available at 10.1007/s00204-021-03185-y.

## Introduction

Next-generation sequencing (NGS) technologies have enabled large-scale genomic mutation analysis and have revealed the role of genomic somatic mutations in human cancer. In recent years, the demand for a precise clarification of genome-wide somatic mutations has increased in various research fields (Kennedy et al. 2012; Beckman and Loeb 2017). In particular, in the field of chemical mutagenicity, direct, genome-wide analysis of mutagen-induced rare mutations has opened opportunities to characterize mutation spectra induced by mutagens. These studies will improve our knowledge of their mechanisms of action and their relationship with carcinogenicity (Maslov et al. 2015; Sloan et al. 2018; Kucab et al. 2019; Salk and Kennedy 2020).

However, standard NGS analysis does not accurately identify rare somatic mutations, which are mainly caused by DNA damage, such as DNA oxidation occurring during DNA shearing, due to the presence of PCR errors (Costello et al. 2013). Duplex consensus sequencing strategies (DCSSs) have been used to correct these sequencing artifacts (Schmitt et al. 2012; Hoang et al. 2016; Salk et al. 2018; Matsumura et al. 2019). Because DNA damage exists on only one strand of the dsDNA fragment, DCSSs can dramatically improve sequencing accuracy (10^−7^–10^−8^ bp) by utilizing sequence information from both strands of dsDNA. We have developed a DCSS called Hawk-Seq™ and demonstrated its use in evaluating chemical mutagenicity and carcinogenicity (Matsumura et al. 2019; Otsubo et al. 2021).

**Fig. 1:**
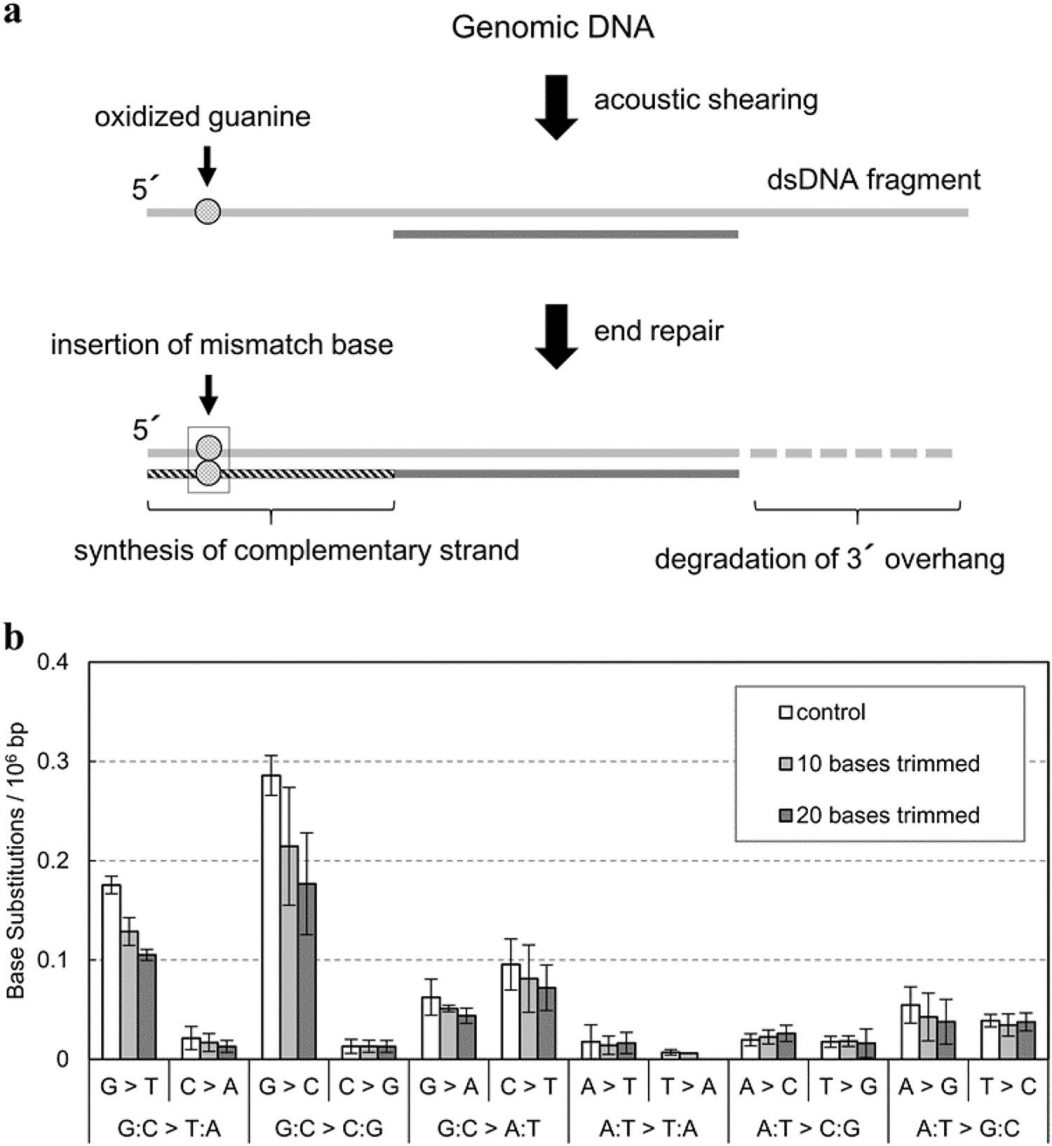
Analysis of the read clipping approach on G:C error reduction. **a** Principle of sequencing error caused by end repair. **b** Error reduction in 12 base substitution types in DMSO-exposed TA100 cells through computational-read trimming. BS frequencies per 10^6^ G, C, A, or T are displayed (*n* = 3). Error bars represent standard deviation

However, some DCSSs studies have indicated the presence of residual errors, especially on G:C base pairs (Kennedy et al. 2014; Peng et al. 2019; You et al. 2020). Using Hawk-Seq™ analysis, we found that the errors on G:C base pairs occurred approximately five times more frequently than those on A:T base pairs. These errors could hamper the detection or characterization of extremely rare mutations (Otsubo et al. 2021). Some have suggested that these errors are attributable to oxidized guanine located on single-strand (SS) overhangs, which may arise at the ends of sonicated DNA fragments. Because the SS overhang regions are repaired using the complementary strand as a template during end-repair, even DCSSs cannot eliminate PCR errors in these regions (Fig. 1a). In addition, these SS regions are vulnerable to DNA damage, such as oxidation or deamination, which may further increase the error frequency, for example, during DNA sample storage. The establishment of a methodology that effectively removes SS-related errors will be useful for precisely characterizing mutations by mutagens (Kavli et al. 2007; Anindya 2020).

SS regions are believed to exist as short overhangs of several bases on both ends of the DNA fragment and can be removed by computationally clipping several bases corresponding to each end of the DNA fragment. For example, Kennedy et al. suggested clipping five bases to remove these artifacts (Kennedy et al. 2014). You et al. clarified that these artifacts are prominent at the terminal 7 base pairs of the DNA fragments (You et al. 2020). However, none of these studies demonstrated that clipping several terminal bases can remove all errors. Therefore, it is unclear whether these read clipping procedures are truly effective in reducing SS overhang-related errors. SS regions might not only be located as short overhangs on terminal regions, but also expand toward the middle of the DNA fragment. In this case, the read clipping approach merely decreases the amount of data without substantial error reduction. Therefore, it is necessary to evaluate the utility of this approach and establish a method that can effectively reduce SS-related errors.

In this study, we first evaluated the effect of computational-read clippings on G:C error reduction up to a length of 20 bp. As these did not sufficiently reduce G:C errors, to remove SS regions more effectively, we evaluated the utility of single-strand specific nucleases (SSNs) by treating them with DNA fragments ahead of the end-repair step. We compared the error reduction ability of three mechanistically distinct SSNs, including S1 Nuclease, mung bean nuclease (MBN), and RecJf exonuclease (RecJf), and considered the status of SS regions based on their behaviors. We also identified the most suitable enzyme (S1 Nuclease) and named this improved sequencing method “Jade-Seq™”. It is an acronym which stands for “Justifies Analyte Dna sEquence”. Finally, the improvement in detection sensitivity was assessed through the analysis of mutagen-exposed DNA samples from *Salmonella typhimurium* TA100 cells.

## Materials and methods

### Preparation of DNA fragment and SSN treatment

Genomic DNA samples of TA100, which were exposed to 1000 µg/tube of 3-methylcholanthrene (3MC), 1000 µg/tube of 7,12-dimethylbenz[a]anthracene (DMBA), and solvent control (DMSO) under suspension culture conditions, were used (Otsubo et al. 2021). First, 60–120 ng of TA100 genomic DNA samples were sheared to fragments with a peak size of 350 bp using a sonicator (Covaris, MA, USA). To remove SS overhangs, the resultant DNA fragments were treated with SSNs, namely S1 Nuclease (Promega Corporation, Madison, WI, USA), MBN (Takara Bio Inc., Shiga, Japan), and RecJf (New England BioLabs, Ipswich, MA, USA). As for the S1 Nuclease treatment, DNA fragments were incubated for 30 min at 30 °C in 40 µl of reaction solution (50 mM sodium acetate (pH 4.5), 0.3 M NaCl, and 4.5 mM ZnSO_4_) containing 1, 3, 10, 30, 100, 300, and 1000 units of S1 Nuclease. Next, to stop the reaction, the solution was mixed with 3 µl of 0.5 M EDTA (Nippon Gene Co., Ltd., Tokyo, Japan) and incubated for 10 min at 70 °C. Regarding the MBN treatment, DNA fragments were incubated for 10 min at 37 °C in 50 µl of reaction solution (30 mM sodium acetate (pH 5.0), 0.1 M NaCl, 1.0 mM zinc acetate, and 5.0% glycerol) containing 3, 10, 30, and 100 units of MBN. Next, the solution was mixed with 3 µl of 0.5 M EDTA and incubated for 10 min at 65 °C. For the RecJf treatment, the DNA fragments were incubated for 1 h at 37 °C in 50 µl of reaction solution (10 mM Tris–HCl (pH 7.9), 50 mM NaCl, 10 mM MgCl_2_, and 1 mM DTT) containing 3, 10, 30, and 100 units of RecJf, followed by incubation for 20 min at 65 °C.

### Library preparation and sequencing

The obtained DNA fragments were used for sequence library preparation using the TruSeq Nano-DNA Library Preparation Kit (TruSeq; Illumina, San Diego, CA, USA), with a slight modification for Hawk-Seq™. Briefly, DNA fragments were subjected to end repair, 3ʹ dA-tailing, and ligation to TruSeq-indexed adaptors, according to the manufacturer’s instructions. Thereafter, the DNA concentration of each ligated sample was measured using the Agilent 4200 TapeStation (Agilent Technologies, CA, USA). Regarding SSN-treated samples, these ligated products were diluted with suspension buffer, and 156, 78, 39, and 20 amol of ligated products were subjected to PCR amplification. The amplified PCR products were sequenced with 2 × 100 bp or 2 × 150 bp to yield ~ 50 M read pairs using HiSeq2500 or HiSeqX (Illumina, San Diego, CA, USA).

### Data processing for Hawk-Seq™

Adaptor sequences and low-quality bases were eliminated from the generated read pairs using Cutadapt-1.16 (Martin 2011). Quality checks of the resulting paired-end reads were conducted using FastQC-0.11.7 (https://www.bioinformatics.babraham.ac.uk/projects/fastqc/. Accessed 12 July 2021), and the proportion of each of the four bases at each base position in the sequenced reads were calculated. The resulting paired-end reads were aligned to the *S. typhimurium* LT-2 genome (GCA000006945.2) as the reference genome sequence to prepare an SAM file using Bowtie2-2.3.4.1 (Langmead et al. 2009). SAM processing was performed using SAMtools-1.7 (Li et al. 2009). The double-strand DNA consensus sequence (dsDCS) was generated according to the Hawk-Seq™ method (Matsumura et al. 2019). Briefly, read pairs that shared the same genomic location were grouped into the same position groups (SP-Gs) and divided into two sub-groups based on their orientation. SP-Gs that included read pairs in both read directions were used to generate dsDCS read pairs. These dsDCS read pairs were mapped again to the reference genome sequence using Bowtie2.

### Mutation detection and statistical analysis

The resulting SAM files were processed using SAMtools for detecting mutations. To evaluate the effect on error reduction of computational-read trimming, the first 10 or 20 bases of each read in the SAM files of DMSO-exposed samples were clipped and subjected to mutation analysis. To calculate BS frequency, the number of base substitutions for each type (i.e., 6-type) was enumerated. Then, BS frequencies for each mutation type per 10^6^ G:C or A:T base pairs were calculated by dividing the mutation count by the total read base count mapped to G:C or A:T base pairs. To evaluate the strand specificity of mutation frequency, each base substitution frequency was separately calculated depending on the base each read base was mapped to (e.g., G or C for the G:C base pair). Statistical analyses were performed based on the frequency of each mutation type per 10^6^ bp using Dunnett’s multiple comparison test or Student’s *t* test. Known variant positions of TA100 were removed from the analysis (Matsumura et al. 2017). To evaluate improvement in detection sensitivity for mutations, the logarithm of fold change of each substitution type in mutagen-exposed samples to base 2 [log_2_ (fold change)] and their negative logarithm of *P* value to base 10 [− log_10_ (*P* value)] were determined.

### Calculation of coverage for reference sequence

Coverage information of dsDCS read pairs on the *S. typhimurium* LT-2 genome was calculated using the pileup format created from SAM files. Then, coverage histograms were created by dividing the LT-2 genome into 50,000 sections. In addition, we calculated the genome coverage rate, which represents the rate of the genomic position at which at least one dsDCS base was mapped. Mean coverage, standard deviation (SD) of coverage, and coefficient of variation (CV) were also determined.

## Results

### Error reduction by computational-read clipping approach

To confirm that the errors on the G:C base pair originated from the SS regions (Fig. 1a), we first evaluated the strand specificity of the six types of BSs (i.e., calculated 12 types of BS frequencies) in DMSO-exposed samples through Hawk-Seq™ analysis. Mean frequencies of each BS on G:C base pairs (*n* = 3) were 0.18 × 10^−6^ bp on G > T, 0.021 × 10^−6^ bp on C > A, 0.28 × 10^−6^ bp on G > C, 0.013 × 10^−6^ bp on C > G, 0.062 × 10^−6^ bp on G > A, and 0.096 × 10^−6^ bp on C > A (Fig. 1b, blank bar; Supplementary Table S1, control column for raw data). The frequencies of these patterns on the 6-type basis were 0.10 × 10^−6^ bp for the G:C > T:A mutation, 0.15 × 10^−6^ bp for the G:C > C:G mutation, and 0.08 × 10^−6^ bp for the G:C > A:T mutation. G > T and G > C errors occurred much more frequently than their counterpart C > A and C > G errors, respectively. These results indicate that errors on G:C base pairs are possibly caused by the artificial modification of the G base.

Then, we trimmed 10 or 20 read bases corresponding to both ends of the DNA fragments by processing sequencing data of DMSO-treated samples and calculated BS frequency (Fig. 1b, Supplementary Table S1 for raw data). We found that frequencies of G > T and G > C mutations decreased according to the length of trimming. When 20 bases were clipped, the G > T and G > C substitution frequencies dropped to 0.11 × 10^−6^ bp and 0.18 × 10^−6^ bp, respectively. Here, the G:C > T:A and G:C > C:G frequencies decreased to 0.059 × 10^−6^ bp and 0.095 × 10^−6^ bp, respectively. However, although significant reductions in error frequencies were observed by read trimmings, G > T and G > C error frequencies were higher than those of their counterpart errors (i.e., C > A and C > G, respectively). Therefore, SS-related errors probably remained even after read trimming. Although further read trimming would lower these error frequencies, it would also substantially decrease the number of bases available for mutation analysis.

### Error reduction using SSNs

**Fig. 2:**
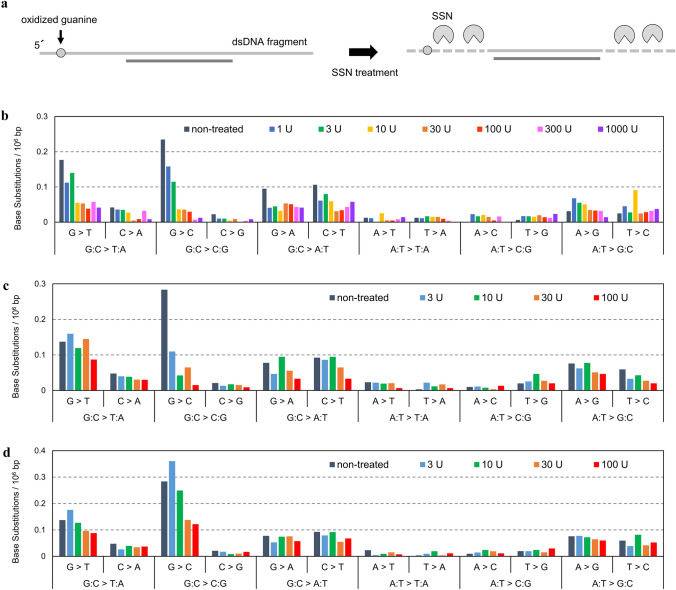
Effectiveness of SSN treatment on error reduction. **a** Principle of SS-related error reduction by SSN treatment. Twelve base substitution types in DMSO-exposed TA100 cells by **b** S1 Nuclease (1, 3, 10, 30, 100, 300, and 1000 U), **c** MBN (3, 10, 30, and 100 U), and **d** RecJf (3, 10, 30, and 100 U). BS frequencies per 10^6^ G, C, A, or T are displayed (*n* = 1)

To overcome these problems, we utilized SSNs to enzymatically remove SS regions after DNA shearing and evaluated their ability to reduce SS-related errors (Fig. 2a). We investigated the reduction in BS frequency in DMSO-exposed samples using three SSNs, namely two endonucleases (SS-endonucleases: S1 Nuclease and MBN) and one exonuclease (SS-exonuclease: RecJf). In S1 Nuclease-treated samples, the G > T and G > C frequencies declined dramatically (Fig. 2b, Supplementary Table S2a for raw data). When using 10 units (U) of S1 Nuclease, G > T and G > C frequencies were 0.055 × 10^−6^ bp and 0.037 × 10^−6^ bp, respectively. Correspondingly, G:C > T:A and G:C > C:G frequencies decreased to 0.041 × 10^−6^ bp and 0.021 × 10^−6^ bp, respectively. In samples treated with ≥ 10 U of S1 Nuclease, G > T and G > C frequencies became almost equivalent to C > A and C > G frequencies, respectively. These results suggest that the errors derived from the SS regions were mostly removed by S1 Nuclease treatment.

In MBN-treated samples, G > T and G > C frequencies also decreased significantly (Fig. 2c, Supplementary Table S2b for raw data). Notably, in samples treated with ≥ 10 U of MBN, G > C frequencies decreased to an almost equivalent level to that of S1 Nuclease-treated samples. The G > C and G:C > C:G frequencies in 10 U of MBN-treated samples were 0.042 × 10^−6^ bp and 0.030 × 10^−6^ bp, respectively. However, although the G > T frequency decreased, it was higher than that in the S1 Nuclease-treated samples. The frequency dropped only to 0.087 × 10^−6^ bp, even in the sample treated with the maximum dose (100 U). The G:C > T:A frequency in this sample was 0.058 × 10^−6^ bp.

After RecJf treatment, G > T and G > C frequencies decreased to 0.088 × 10^−6^ bp and 0.12 × 10^−6^ bp, respectively (Fig. 2d, Supplementary Table S2c for raw data). In this sample, the G:C > T:A and G:C > C:G frequencies were 0.063 × 10^−6^ bp and 0.069 × 10^−6^ bp, respectively. These values were substantially lower than those obtained after computational trimming of 20 bp. However, these were higher than those in S1 Nuclease- or MBN-treated samples.

Although the frequencies of G > A and C > T mutations decreased in S1 Nuclease- and MBN-treated samples, the difference was not significant compared to the reduction in G > T and G > C frequencies.

Overall, all three SSNs reduced the G > T and G > C frequencies more effectively than computational-read trimming. Among the three SSNs, the S1 Nuclease decreased the error frequencies most effectively. In addition, these results strongly indicate that the remaining errors on the G bases are derived from the SS regions.

### Influence on genome coverage and sequence specificity by SSNs

**Fig. 3:**
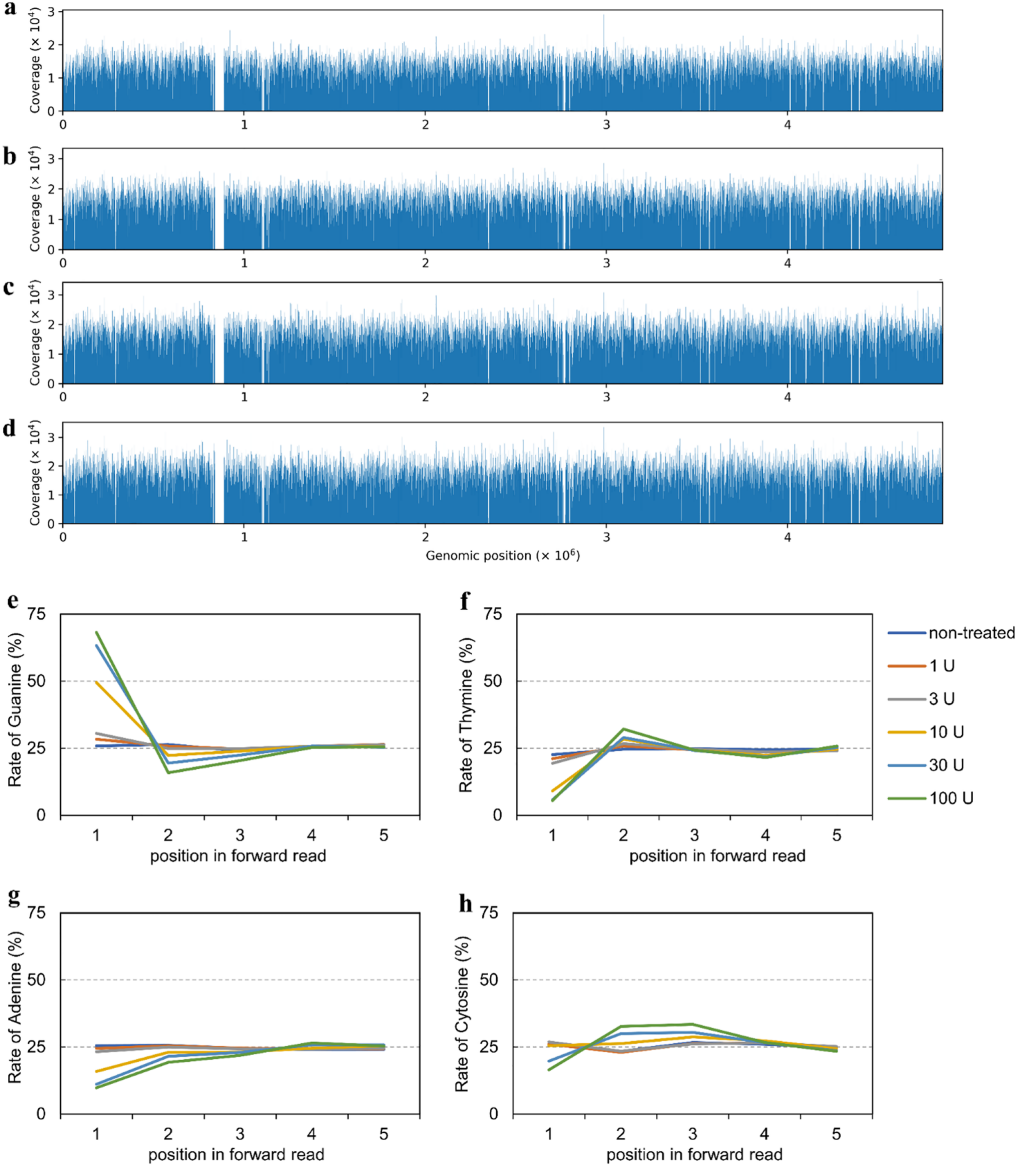
Effects of S1 Nuclease treatment on genome coverage and bias of read sequence. Histograms of genome coverage are shown for **a** non-treated and S1 Nuclease-treated (**b** 10 U, **c** 30 U, and **d** 100 U) DNA samples. Effects of S1 Nuclease treatment (1, 3, 10, 30, and 100 U) on proportion of each of the four normal DNA bases (**e** G, **f** T, **g** A, and h C) at the first five bases in forward reads

Because nucleases commonly have sequence specificity, there is a possibility that they may cause sequence bias, which could influence the overall mutation landscape characterized by DCSSs analysis. Therefore, we evaluated the effects on overall coverage of the *Salmonella* genome and on read-sequence specificity using Hawk-Seq™ analysis. Figure 3a–d and Supplementary Figure S1a and b show the distributions of the number of bases mapped to each region of the LT-2 genome under SSN treatment. These results indicated that there was no clear sequence bias due to SSN treatments (Fig. 3a–d, Supplementary Fig. S1a, b). Furthermore, we confirmed that the rate of genomic region covered by at least one DCS base (%) and its CV were not affected (Table 1). These results suggest that SSN treatments have little effect on overall genome coverage and, consequently, on overall mutation landscapes under DCSSs analysis.

**Table 1:** Effects of SSNs on parameters for genome coverage

SSNs (100 U)	Covered rate (%)	Coverage		
		Mean	SD	CV
Non-treated	97.5	141	37.1	0.264
S1 Nuclease	97.5	185	48.6	0.263
MBN	97.5	262	67.6	0.258
RecJf	97.5	232	59.5	0.257

Representative data from samples treated with 100 U of S1 Nuclease, MBN, and RecJf (*n* = 1)

*CV* coefficient of variation, *MBN* mung bean nuclease, *SD* standard deviation, *SSN* SS-specific nuclease

We then evaluated the occurrence of bias in the read sequence due to SSN treatment. We found a bias within the first ~ 5 bases in both forward and reverse reads with both S1 Nuclease and MBN treatments (Fig. 3e–h, Supplementary Fig. S1c–f). In S1 Nuclease-treated samples, the ratio of G at the first base increased according to the concentration of the S1 Nuclease. Meanwhile, the ratio of G and C bases at the first several bases increased depending on the concentration of MBN. These biases are probably caused by the sequence preferences of SSNs. DCSS analyses do not utilize molecular barcodes, such as Hawk-Seq™; this could increase the number of DNA fragments with identical genomic positions, thereby increasing the incidence of oversight of true mutations (Matsumura et al. 2019). Indeed, S1 Nuclease and MBN treatment increased the rate of SP-Gs that contained read pairs originating from different dsDNA fragments (Supplementary Fig. S2a, b). The possibility of this phenomenon could be minimized by reducing the amount of ligated products (Supplementary Fig. S2d). In contrast to S1 Nuclease and MBN, RecJf treatment did not indicate significant sequence bias in the terminal bases of the fragment (Supplementary Fig. S1g–j). Accordingly, the ratio of SP-Gs, including misassigned read pairs, did not increase with RecJf treatment (Supplementary Fig. S2c).

### Application of Jade-seq™ to mutagen-induced mutation analysis

**Fig. 4:**
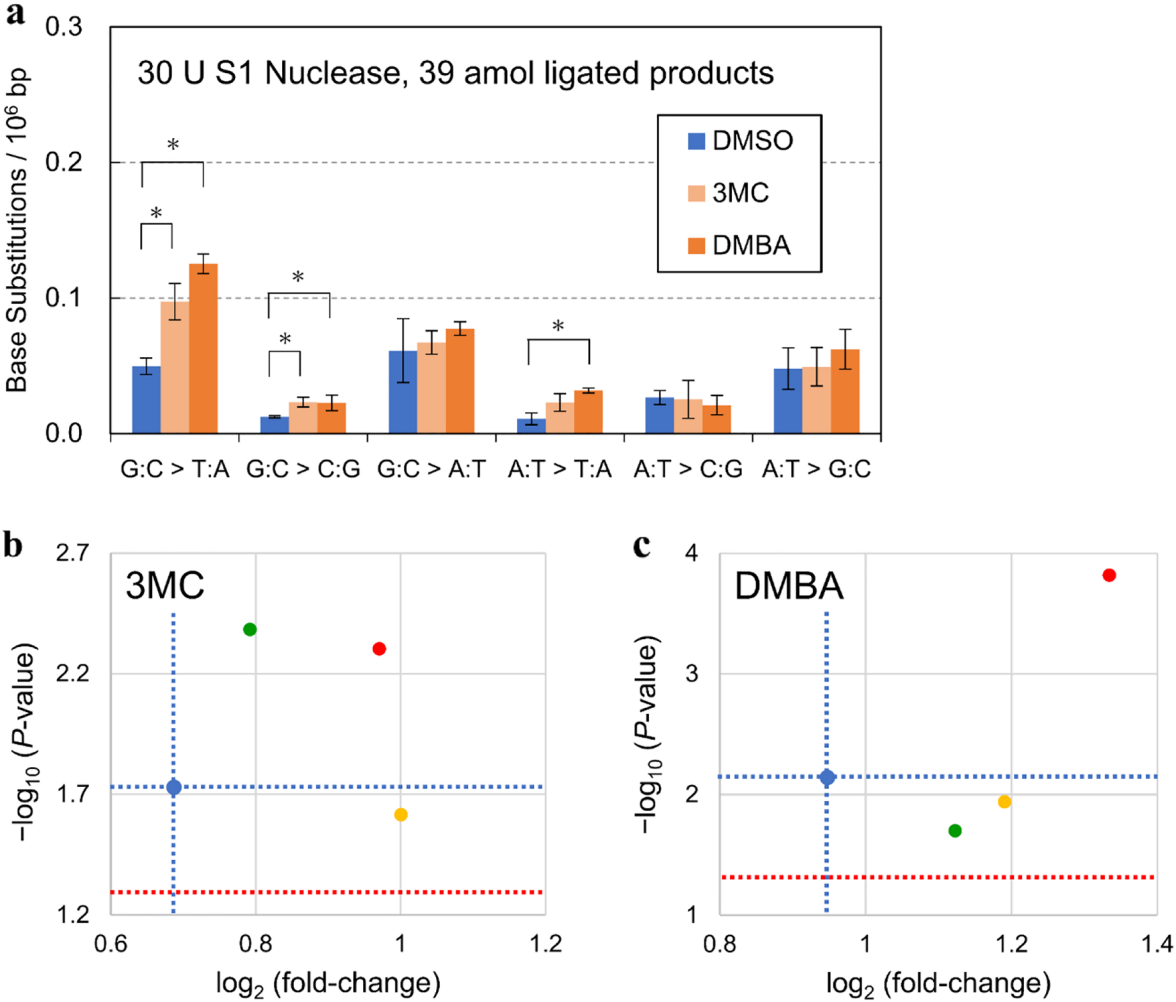
Enhancement of detection sensitivity for 3MC- or DMBA-induced mutations through S1 Nuclease treatment. Libraries were prepared with 78, 39, and 20 amol of ligated products using DNA samples treated with 10 and 30 U of S1 Nuclease. **a** Representative result of mutation pattern induced by DMSO, 3MC (1000 µg/tube), or DMBA (1000 µg/tube) in TA100 cells (S1 Nuclease, 30 U; ligated products, 39 amol). BS frequencies per 10^6^ G:C or A:T base pairs are displayed (*n* = 3). Error bars represent standard deviation. *P < 0.05 by Student’s *t* test. [log_2_ (fold change)] and [− log_10_ (*P* value)] of G:C > T:A mutation in **b** 3MC- or **c** DMBA-exposed samples treated with 30 U of S1 Nuclease under different conditions of the ligated products (yellow, 78 amol; red, 39 amol; green, 20 amol) and non-treated samples (blue: 0 U of S1 Nuclease and 78 amol of ligated products) were calculated (*n* = 3). Blue broken lines indicate the values of non-treated samples exposed to each mutagen. Red broken lines represent [− log_10_ (*P* value)] when *P* value is 0.05

We named our new error-corrected sequence technology, which utilizes the S1 Nuclease, Jade-Seq™. Next, we evaluated the improvement in mutation-detection sensitivity using Jade-Seq™ analysis in comparison with Hawk-Seq™ analysis. We analyzed 3MC- and DMBA-induced mutations treated with 30 U of S1 Nuclease under various amount of ligated products (amol). As the genomic DNA samples that were exposed to 3MC and DMBA showed only a slight increase (approximately 2 × 10^−7^ bp) in mutation frequencies compared to DMSO controls, we used as model samples of ultra-rare mutations. For DMSO-exposed samples, the error frequencies, without S1 Nuclease treatment, on G:C > T:A and G:C > C:G were 0.11 × 10^−6^ bp and 0.16 × 10^−6^ bp, respectively (Supplementary Fig. S3a, Supplementary Table S3a for raw data). These errors were decreased by S1 Nuclease treatment (Fig. 4a, Supplementary Fig. S3b, c, Supplementary Table S3b-d for raw data), to 0.050 × 10^−6^ bp and 0.012 × 10^−6^ bp, respectively (Fig. 4a). In 3MC- and DMBA-exposed samples, clear increases in the frequency of G:C > T:A mutation, a major mutation pattern by these mutagens (Gorelick et al. 1995; Rihn et al. 2000), were detected in both S1 Nuclease-treated and non-treated samples. Specifically, regarding 3MC-induced G:C > T:A mutations, the fold changes and their negative log of *P* values were both increased by S1 Nuclease treatment (Fig. 4b). Meanwhile, in DMBA-exposed samples (Fig. 4c), the fold changes of G:C > T:A mutations increased by S1 Nuclease treatment, indicating an improvement in detection sensitivity.

## Discussion

Our findings reveal that treatments with SSNs, especially Jade-Seq™, reduce SS-related errors more effectively than terminal-base clipping of DNA fragments, causing little effect on DCSS performance. Therefore, Jade-Seq™ would enable the sensitive characterization of rare, mutagen-induced mutations.

Of the SSNs evaluated, the SS-endonucleases S1 Nuclease and MBN were more effective in reducing G > T and G > C errors than the SS-exonuclease RecJf. These results suggest that SS overhangs formed after acoustic shearing extended longer than expected (Kennedy et al. 2014; You et al. 2020). In addition, SS regions might not only exist as short overhangs in the terminal region, but also as gaps in the middle of the fragment. SS-endonucleases would be more effective in error reduction, because these enzymes can more effectively degrade SS regions.

When the two SS-endonucleases were compared, S1 Nuclease was more effective in G > T error reduction than MBN. We speculate that this is attributable to the difference in the activity to nicks between these two enzymes (Supplementary Fig. S4). As shown in Supplementary Fig. S4, the right side (i.e., the 3ʹ end) of the dsDNA region, after nicked sites, might act like pseudo-dsDNA, because the DNA strand in these DNA regions would be replaced in the 5ʹ to 3ʹ direction during the end-repair process. As the S1 Nuclease can effectively catalyze nicked sites more effectively than MBN, this enzyme might more effectively prevent contamination of nicked DNA fragments into the sequence library. Thus, S1 Nuclease, which has wide substrate specificity, is the most suitable enzyme for reducing SS-related errors.

Contrary to G > T errors, G > C errors were almost equivalently eliminated by MBN and S1 Nuclease. We consider that this discrepancy is due to the difference in the origins of DNA damage causing these two errors. G > T errors are caused by 7,8-dihydro-8-oxoguanine (8-oxoG) in NGS analysis (Costello et al. 2013). Meanwhile, the origin of G > C errors in NGS analysis has not been clarified. However, 2-aminoimidazolone (Iz), known as oxidized G, induces G:C > C:G mutations (Kino and Sugiyama 2001; Neeley et al. 2004). In addition, a few studies have reported that guanines are mainly transformed into Iz in SS DNA under photooxidative conditions (Morikawa et al. 2013, 2014). Therefore, although it is unclear whether Iz also occurs during NGS library preparation, G > C transversion might be induced more frequently in naked (not-sealed) SS DNA regions. Therefore, these two SS-endonucleases, which eliminate these SS regions, might have sufficiently removed G > C errors.

In Jade-Seq™ analysis, G:C > T:A and G:C > C:G error frequencies decreased to 0.050 × 10^−6^ bp and 0.012 × 10^−6^ bp, respectively. Indeed, various human carcinogens, such as benzo[a]pyrene (BP), induce G:C > T:A mutations. For example, BP induced G:C > T:A mutation at the frequencies of 0.5 × 10^−6^ bp in bacteria, 1.2 × 10^−6^ bp in vivo in mouse (Matsumura et al. 2019), and ~ 0.3 × 10^−6^ bp in vitro in human cells (Kucab et al. 2019). Therefore, Jade-Seq™ would be useful for the extensive analysis of mutations. Matsuda et al. reported that the spontaneous mutation frequency in bacteria would be 0.046 × 10^−6^ bp, based on the data acquired from genome samples from several revertant colonies of TA100 (Matsuda et al. 2013). Although the amount of data regarding spontaneous mutation frequency has been limited owing to its rarity, these reports suggest that Jade-Seq™ could be useful in clarifying them.

Moreover, SSN treatment could be useful for the analysis of DNA samples, which would have a considerable number of SS regions or oxidative damage. For example, in short DNA fragments that are used for capture-based targeted sequencing, G:C > T:A and G:C > C:G errors were observed more frequently, probably because stronger DNA shearing promotes guanine oxidation and generation of SS regions (Park et al. 2017). DNA samples from formalin-fixed tissues contain various DNA lesions, such as abasic sites, cytosine deamination, and single-strand breaks, which lead to dsDNA denaturation and SS region generation (Do and Dobrovic 2015). A recent investigation suggested that cell-free DNA might carry longer SS overhangs than sonicated DNA fragments (Jiang et al. 2020). Therefore, Jade-Seq™ would be useful for the analysis of preclinical and clinical DNA samples by reducing SS-related errors in these samples.

In conclusion, we established a novel method to reduce SS-related G:C > T:A and G:C > C:G errors using SSNs. We also gained insights into sequence error mechanisms based on the relationship between the error reduction abilities and the mechanisms of action of enzymes. These findings would enable effective error reduction, which is essential for enhancing the detection sensitivity for extremely rare mutations.

## Supplementary Information

Below is the link to the electronic supplementary material.

Supplementary file1 (DOCX 966 KB)

## Data Availability

Sequence data of the *S. typhimurium* strain used in this study are available from the DNA Data Bank of Japan Sequence Read Archive under accession number DRA012398.
