# Supplementary material for: Single-strand specific nuclease enhances accuracy of error-corrected sequencing and improves rare mutation-detection sensitivity
Source: Arch Toxicol. 2021 Nov 12;96(1):377–86. doi: 10.1007/s00204-021-03185-y (PMC8748355; doi:10.1007/s00204-021-03185-y)
Supplement: Supplementary file 1 — Supplementary file1 (DOCX 966 KB) [file 204_2021_3185_MOESM1_ESM.docx]

Supplementary data

*Archives of Toxicology*

**Single-strand specific nuclease enhances accuracy of error-corrected sequencing and improves rare mutation detection sensitivity**

Yuki Otsubo^1^, Shoji Matsumura^1,*^, Naohiro Ikeda^1^ and Masayuki Yamane^2^

^1^R&D Safety Science Research, Kao Corporation, 3-25-14 Tono-machi, Kawasaki-ku, Kawasaki City, Kanagawa 210-0821, Japan

^2^R&D Safety Science Research, Kao Corporation, 2606 Akabane, Ichikai-Machi, Haga-Gun, Tochigi 321-3497, Japan

*To whom correspondence should be addressed: Shoji Matsumura.

Tel: +81-70-3301-1852; Fax; +81-285-68-7303; Email: matsumura.shouji@kao.com

**Supplementary Table S1**

Number of dsDCS read bases and mutations in three DMSO-exposed TA100 cells in the analyses of computational trimming.

|  | | | control | 10 bases trimmed | 20 bases trimmed |
| --- | --- | --- | --- | --- | --- |
| dsDCS read base | | Total | 2,331,254,077 | 2,031,805,319 | 1,758,328,180 |
|  |  | G | 603,125,190 | 526,797,634 | 456,762,772 |
|  |  | A | 562,190,915 | 488,782,261 | 422,132,555 |
|  |  | C | 603,614,140 | 527,328,790 | 457,246,661 |
|  |  | T | 562,323,832 | 488,896,634 | 422,186,192 |
| Mutations | G:C>T:A | G>T | 106 | 68 | 48 |
|  |  | C>A | 13 | 9 | 6 |
|  | G:C>C:G | G>C | 172 | 112 | 80 |
|  |  | C>G | 8 | 7 | 6 |
|  | G:C>A:T | G>A | 38 | 27 | 20 |
|  |  | C>T | 58 | 43 | 33 |
|  | A:T>T:A | A>T | 10 | 7 | 7 |
|  |  | T>A | 4 | 3 | 0 |
|  | A:T>C:G | A>C | 11 | 11 | 11 |
|  |  | T>G | 10 | 9 | 7 |
|  | A:T>G:C | A>G | 31 | 21 | 16 |
|  |  | T>C | 22 | 17 | 16 |

**Supplementary Table S2**

Number of dsDCS read bases and mutations in DMSO-exposed TA100 cells under **a** S1 Nuclease (1, 3, 10, 30, 100, 300, and 1000 U), **b** MBN (3, 10, 30, and 100 U), and **c** RecJf (3, 10, 30, and 100 U) treatment (n=1).

**a**

|  | | | non-treated | 1 U | 3 U | 10 U | 30 U | 100 U | 300 U | 1000 U |
| --- | --- | --- | --- | --- | --- | --- | --- | --- | --- | --- |
| dsDCS read base | | Total | 682,357,211 | 747,581,198 | 763,850,794 | 834,598,161 | 859,187,809 | 897,728,847 | 1,060,676,637 | 912,371,721 |
|  |  | G | 179,015,534 | 196,166,302 | 200,386,518 | 218,864,892 | 225,510,510 | 236,295,545 | 279,807,880 | 242,615,185 |
|  |  | A | 162,041,865 | 177,501,527 | 181,433,117 | 198,352,184 | 203,947,711 | 212,436,457 | 250,341,935 | 213,398,405 |
|  |  | C | 179,244,168 | 196,376,235 | 200,593,720 | 219,052,739 | 225,758,873 | 236,606,492 | 280,224,597 | 243,021,855 |
|  |  | T | 162,055,644 | 177,537,134 | 181,437,439 | 198,328,346 | 203,970,715 | 212,390,353 | 250,302,225 | 213,336,276 |
| Mutations | G:C>T:A | G>T | 35 | 22 | 28 | 12 | 12 | 9 | 16 | 10 |
|  |  | C>A | 6 | 7 | 7 | 6 | 1 | 2 | 9 | 2 |
|  | G:C>C:G | G>C | 42 | 31 | 23 | 8 | 8 | 7 | 2 | 3 |
|  |  | C>G | 4 | 2 | 2 | 1 | 2 | 0 | 1 | 2 |
|  | G:C>A:T | G>A | 17 | 8 | 9 | 7 | 12 | 12 | 12 | 10 |
|  |  | C>T | 19 | 12 | 16 | 13 | 7 | 8 | 12 | 14 |
|  | A:T>T:A | A>T | 2 | 2 | 0 | 5 | 1 | 1 | 2 | 3 |
|  |  | T>A | 2 | 2 | 3 | 3 | 3 | 2 | 1 | 0 |
|  | A:T>C:G | A>C | 0 | 4 | 3 | 4 | 3 | 1 | 4 | 0 |
|  |  | T>G | 1 | 3 | 3 | 3 | 4 | 3 | 3 | 5 |
|  | A:T>G:C | A>G | 5 | 12 | 10 | 10 | 7 | 7 | 8 | 3 |
|  |  | T>C | 4 | 8 | 5 | 18 | 5 | 6 | 8 | 8 |

**b**

|  | | | non-treated* | 3 U | 10 U | 30 U | 100 U |
| --- | --- | --- | --- | --- | --- | --- | --- |
| dsDCS read base | | Total | 1,277,742,734 | 1,151,097,326 | 1,086,447,878 | 1,239,700,599 | 1,270,748,943 |
|  |  | G | 335,130,108 | 301,153,262 | 284,946,580 | 324,734,563 | 333,661,819 |
|  |  | A | 303,524,078 | 274,239,070 | 258,099,274 | 294,900,279 | 301,544,357 |
|  |  | C | 335,532,937 | 301,440,131 | 285,245,766 | 325,120,337 | 334,061,067 |
|  |  | T | 303,555,611 | 274,264,863 | 258,156,258 | 294,945,420 | 301,481,700 |
| Mutations | G:C>T:A | G>T | 46 | 48 | 34 | 47 | 29 |
|  |  | C>A | 16 | 12 | 11 | 10 | 10 |
|  | G:C>C:G | G>C | 95 | 33 | 12 | 21 | 5 |
|  |  | C>G | 7 | 4 | 5 | 5 | 3 |
|  | G:C>A:T | G>A | 26 | 14 | 27 | 18 | 11 |
|  |  | C>T | 31 | 26 | 27 | 21 | 11 |
|  | A:T>T:A | A>T | 7 | 6 | 5 | 6 | 2 |
|  |  | T>A | 1 | 6 | 3 | 5 | 2 |
|  | A:T>C:G | A>C | 3 | 3 | 2 | 1 | 4 |
|  |  | T>G | 6 | 7 | 12 | 8 | 6 |
|  | A:T>G:C | A>G | 23 | 17 | 20 | 15 | 14 |
|  |  | T>C | 18 | 9 | 11 | 8 | 6 |

**c**

|  | | | non-treated* | 3 U | 10 U | 30 U | 100 U |
| --- | --- | --- | --- | --- | --- | --- | --- |
| dsDCS read base | | Total | 1,277,742,734 | 868,054,516 | 874,148,967 | 1,109,353,252 | 1,125,229,391 |
|  |  | G | 335,130,108 | 227,481,540 | 228,716,332 | 290,627,622 | 295,355,011 |
|  |  | A | 303,524,078 | 206,438,581 | 208,217,114 | 263,866,129 | 267,050,927 |
|  |  | C | 335,532,937 | 227,732,847 | 228,971,839 | 291,001,497 | 295,691,093 |
|  |  | T | 303,555,611 | 206,401,548 | 208,243,682 | 263,858,004 | 267,132,360 |
| Mutations | G:C>T:A | G>T | 46 | 40 | 29 | 28 | 26 |
|  |  | C>A | 16 | 6 | 9 | 10 | 11 |
|  | G:C>C:G | G>C | 95 | 82 | 57 | 40 | 36 |
|  |  | C>G | 7 | 4 | 2 | 3 | 5 |
|  | G:C>A:T | G>A | 26 | 12 | 17 | 22 | 17 |
|  |  | C>T | 31 | 18 | 21 | 16 | 20 |
|  | A:T>T:A | A>T | 7 | 1 | 2 | 4 | 2 |
|  |  | T>A | 1 | 2 | 4 | 1 | 3 |
|  | A:T>C:G | A>C | 3 | 3 | 5 | 5 | 3 |
|  |  | T>G | 6 | 4 | 5 | 4 | 8 |
|  | A:T>G:C | A>G | 23 | 16 | 15 | 17 | 16 |
|  |  | T>C | 18 | 8 | 17 | 11 | 14 |

* The non-treated control sample was the same for the MBN and RecJf treatments.

**Supplementary Table S3**

Number of dsDCS read bases and mutations in three TA100 samples treated with DMSO, 3MC (1000 µg/tube), or DMBA (1000 µg/tube) (**a** non-treated, 78 amol of ligated products; **b** 30 U of S1 Nuclease, 78 amol of ligated products; **c** 30 U of S1 Nuclease, 39 amol of ligated products; **d** 30 U of S1 Nuclease, 20 amol of ligated products).

**a**

| Chemical | | DMSO | 3MC | DMBA |
| --- | --- | --- | --- | --- |
| dsDCS read base | Total | 2,834,003,102 | 2,737,634,270 | 2,259,750,981 |
|  | G | 742,540,336 | 716,719,991 | 590,473,057 |
|  | A | 673,944,703 | 651,661,772 | 539,103,781 |
|  | C | 743,457,281 | 717,479,665 | 590,920,259 |
|  | T | 674,060,782 | 651,772,842 | 539,253,884 |
| Mutations | G:C>T:A | 158 | 254 | 248 |
|  | G:C>C:G | 234 | 267 | 253 |
|  | G:C>A:T | 155 | 148 | 109 |
|  | A:T>T:A | 21 | 20 | 21 |
|  | A:T>C:G | 22 | 24 | 16 |
|  | A:T>G:C | 76 | 63 | 35 |

**b**

| Chemical | | DMSO | 3MC | DMBA |
| --- | --- | --- | --- | --- |
| dsDCS read base | Total | 2,659,426,182 | 2,879,097,287 | 2,278,242,685 |
|  | G | 696,486,150 | 753,911,480 | 594,990,875 |
|  | A | 632,864,978 | 685,322,985 | 543,888,737 |
|  | C | 697,198,226 | 754,570,800 | 595,468,086 |
|  | T | 632,876,828 | 685,292,022 | 543,894,987 |
| Mutations | G:C>T:A | 70 | 153 | 137 |
|  | G:C>C:G | 25 | 31 | 20 |
|  | G:C>A:T | 67 | 80 | 100 |
|  | A:T>T:A | 20 | 23 | 28 |
|  | A:T>C:G | 15 | 14 | 17 |
|  | A:T>G:C | 42 | 41 | 32 |

**c**

| Chemical | | DMSO | 3MC | DMBA |
| --- | --- | --- | --- | --- |
| dsDCS read base | Total | 4,503,154,556 | 4,214,287,825 | 4,457,017,197 |
|  | G | 1,171,848,199 | 1,095,764,678 | 1,159,665,176 |
|  | A | 1,079,283,651 | 1,011,040,672 | 1,068,620,648 |
|  | C | 1,172,783,336 | 1,096,473,708 | 1,160,178,457 |
|  | T | 1,079,239,370 | 1,011,008,767 | 1,068,552,916 |
| Mutations | G:C>T:A | 116 | 214 | 291 |
|  | G:C>C:G | 29 | 51 | 53 |
|  | G:C>A:T | 144 | 146 | 179 |
|  | A:T>T:A | 24 | 47 | 68 |
|  | A:T>C:G | 58 | 52 | 44 |
|  | A:T>G:C | 103 | 101 | 134 |

**d**

| Chemical | | DMSO | 3MC | DMBA |
| --- | --- | --- | --- | --- |
| dsDCS read base | Total | 2,499,314,974 | 2,323,736,819 | 2,363,141,881 |
|  | G | 649,265,843 | 603,516,940 | 613,770,962 |
|  | A | 600,119,346 | 558,150,599 | 567,619,031 |
|  | C | 649,818,903 | 603,945,195 | 614,080,141 |
|  | T | 600,110,882 | 558,124,085 | 567,671,747 |
| Mutations | G:C>T:A | 76 | 122 | 155 |
|  | G:C>C:G | 39 | 28 | 35 |
|  | G:C>A:T | 107 | 103 | 97 |
|  | A:T>T:A | 32 | 30 | 40 |
|  | A:T>C:G | 39 | 25 | 23 |
|  | A:T>G:C | 90 | 80 | 68 |


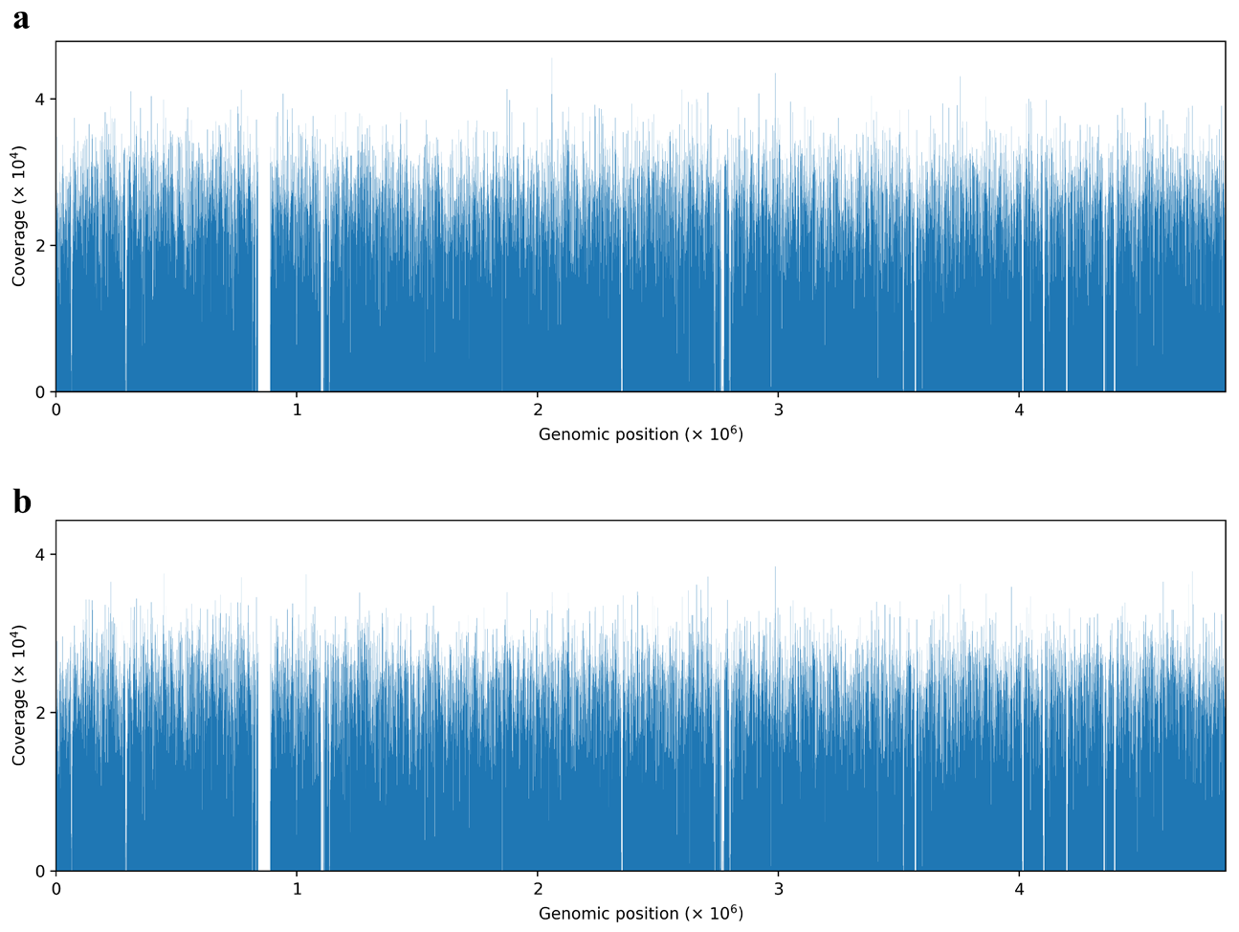


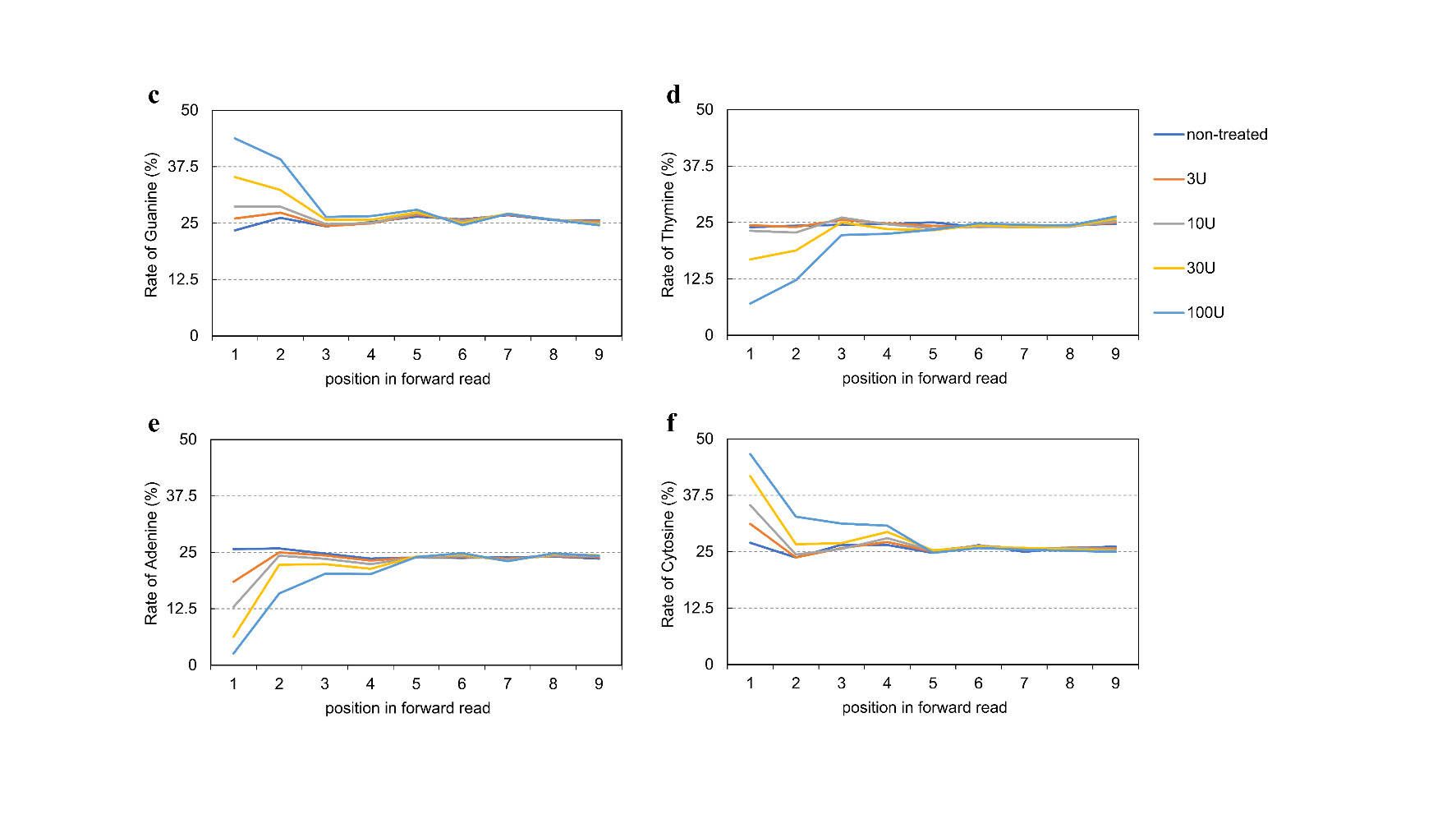


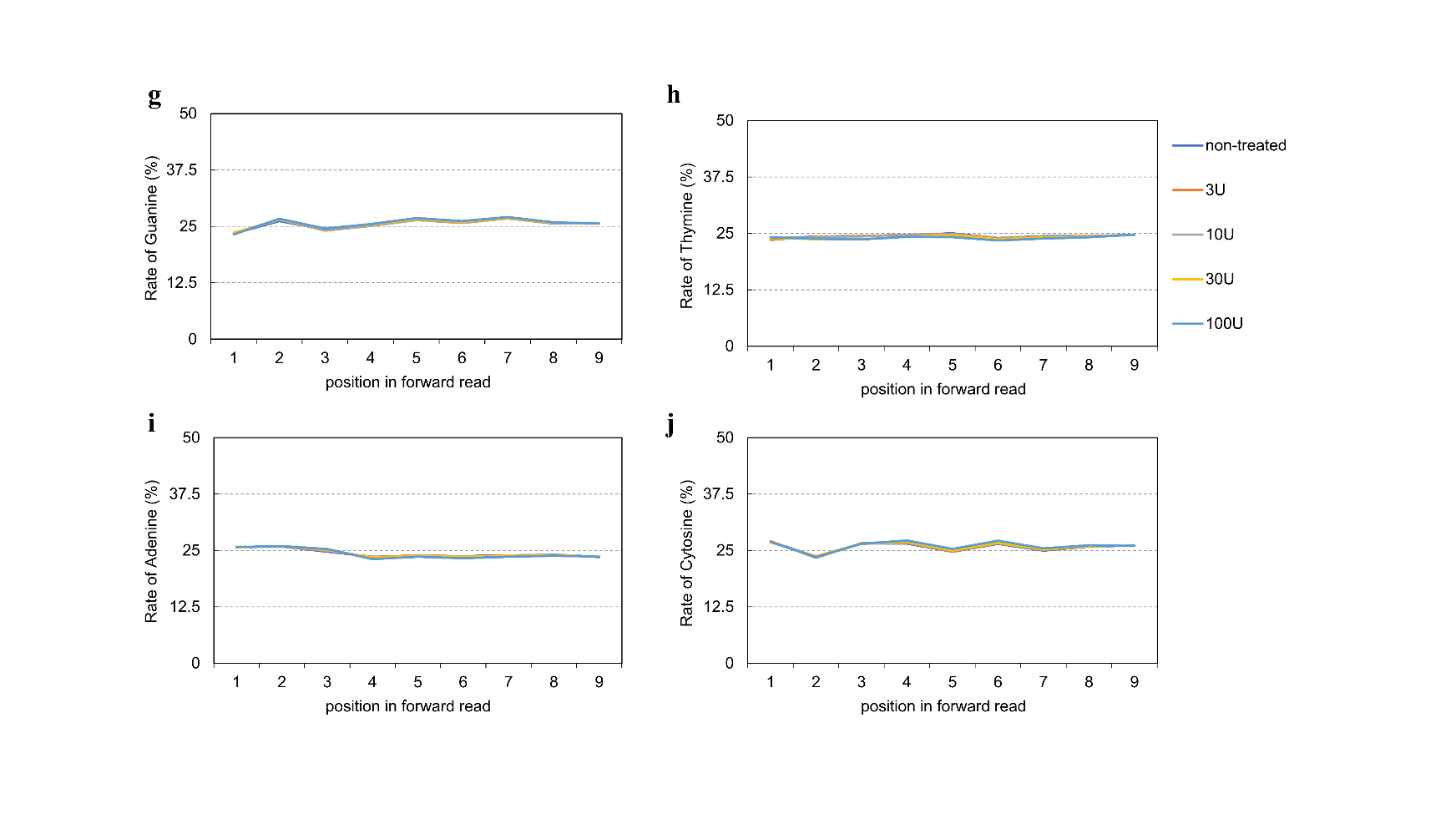


**Supplementary Fig. S1**

Effects of MBN and RecJf treatment on genome coverage and bias of read sequence. Histograms of genome coverage for DNA sample treated with 100 U of **a** MBN and **b** RecJf are shown. Effects of MBN and RecJf treatment (3,10, 30, and 100 U) on proportion of each of the four normal DNA bases (**c, g** G, **d, h** T, **e, i** A and **f, j** C) at the first nine bases in forward read, respectively

**
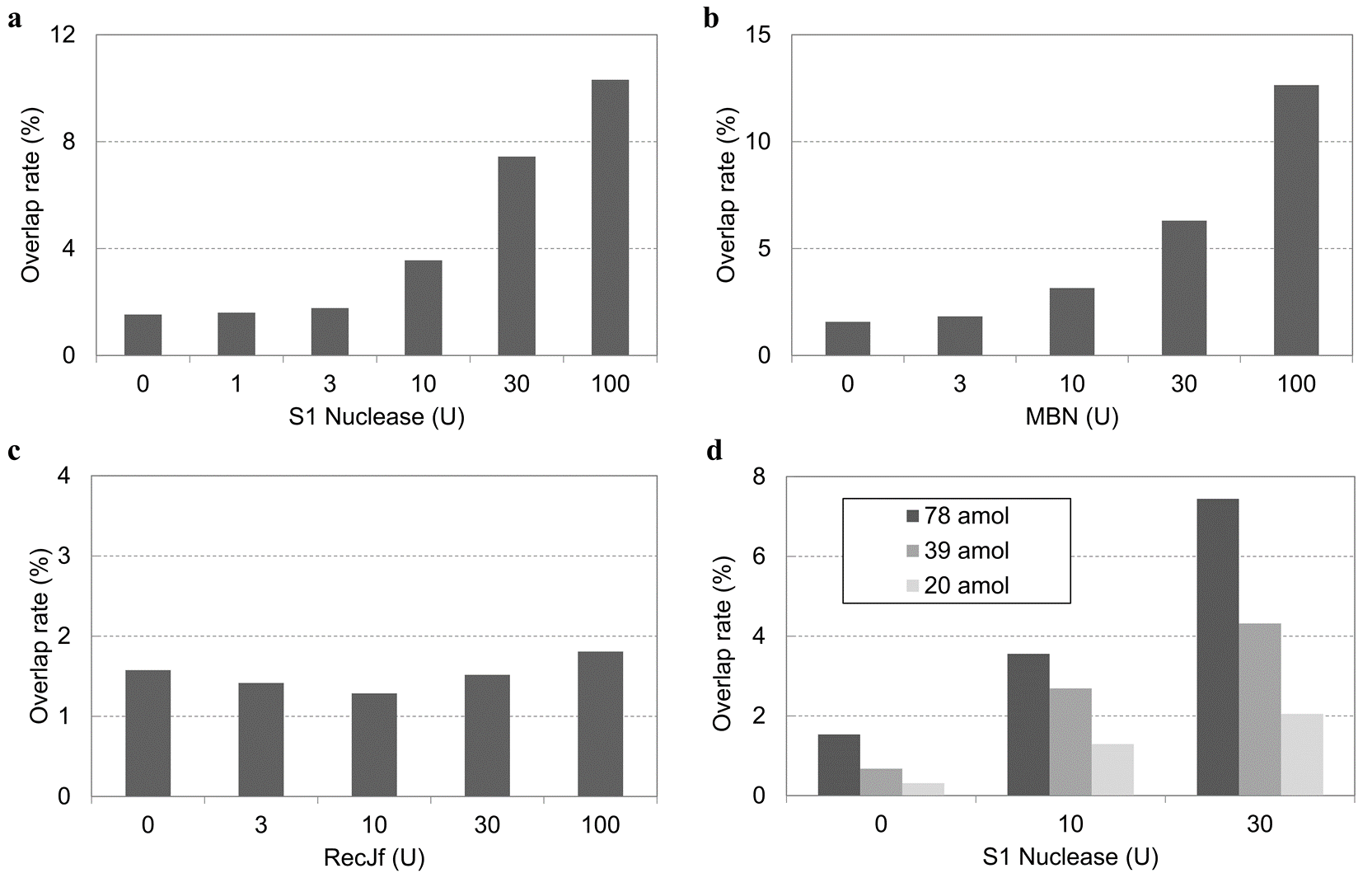
**

**Supplementary Fig. S2**

Because Hawk-Seq™ does not use external molecular barcodes, read pairs originating from the DNA fragment of identical genomic position could be incorrectly assigned into a group to create a dsDCS (Matsumura et al. 2019). We call this phenomenon “overlap by accident (OBA),” which could lead to the underestimation of true mutations. We confirmed the influence of SSNs treatment on OBA probability. To calculate OBA probability, sequencing data created from the DMSO- and mutagen-exposed samples, which have different TruSeq indexes but were prepared under the same condition, were simultaneously mapped to the reference genome sequence. Then, SP-Gs were generated according to the Hawk-Seq™ method. The overlap rate of 2 idxs was calculated by dividing the number of SP-Gs including read pairs of two indexes (SPG-2idxs) by the number of SP-Gs composed of 2 or more read pairs. The overlap rate in **a** S1 Nuclease-, **b** MBN- or **c** RecJf-treated DNA samples is presented (n = 1). The amount of ligated products of these samples were 78 amol. **d** The overlap rate in the S1 Nuclease-treated samples in which the amounts of ligated products were reduced (i.e., 78, 39, and 20 amol, n = 1). Although OBA probability increased by SSN treatment, this could be minimized by controlling the amount of ligated products

**
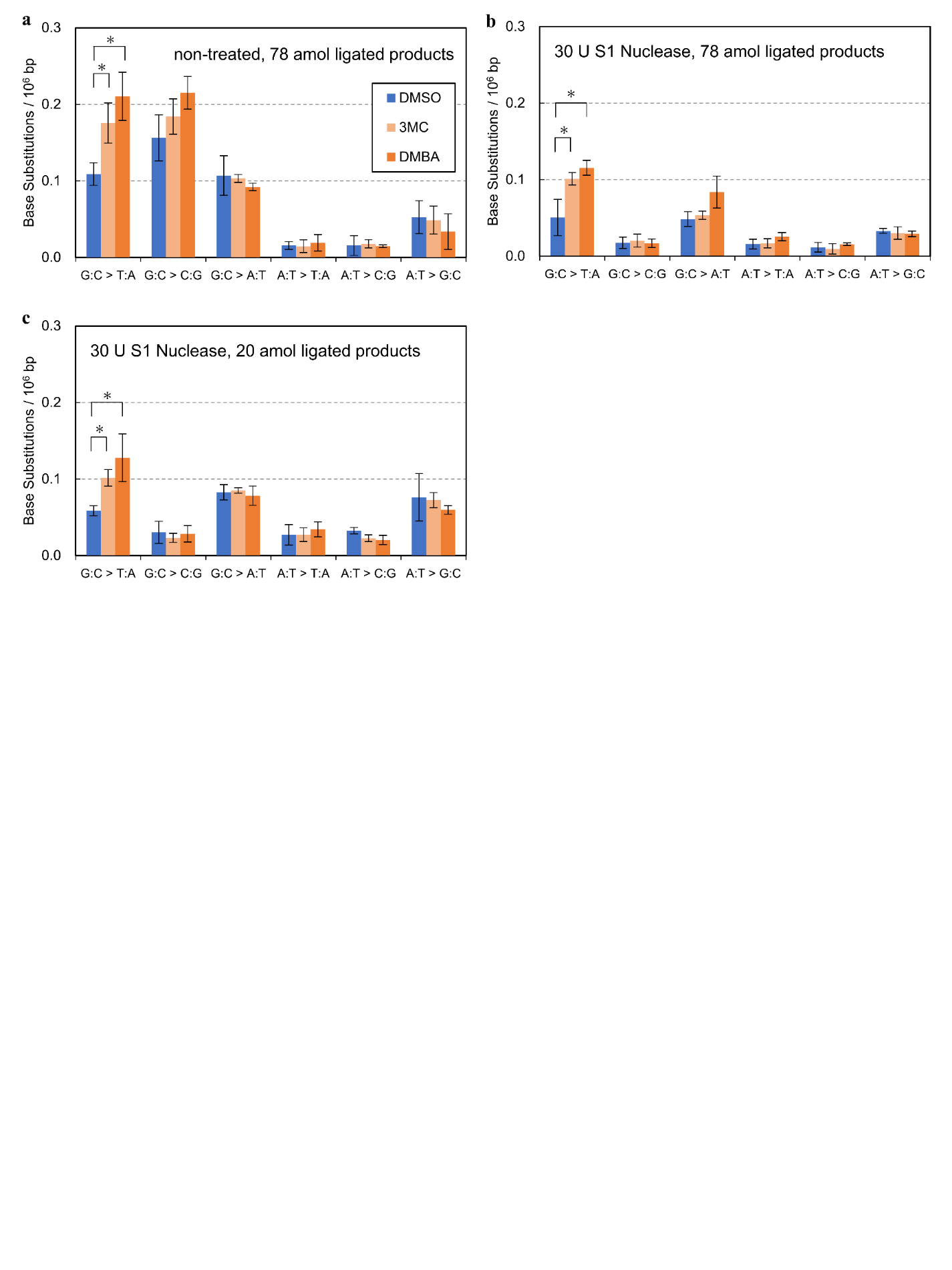
**

**Supplementary Fig. S3**

Enhancement in detection sensitivity of 3MC- or DMBA-induced mutation by S1 Nuclease treatment. Mutation patterns induced by DMSO, 3MC (1000 µg/tube), or DMBA (1000 µg/tube) in TA100 cells (**a** non-treated, 78 amol of ligated products; **b** 30 U of S1 Nuclease, 78 amol of ligated products; **c** 30 U of S1 Nuclease, 20 amol of ligated products). BS frequencies per 10^6^ G:C or A:T base pairs are displayed (n = 3). Error bars represent standard deviation. **P* < 0.05 using Student’s *t* test


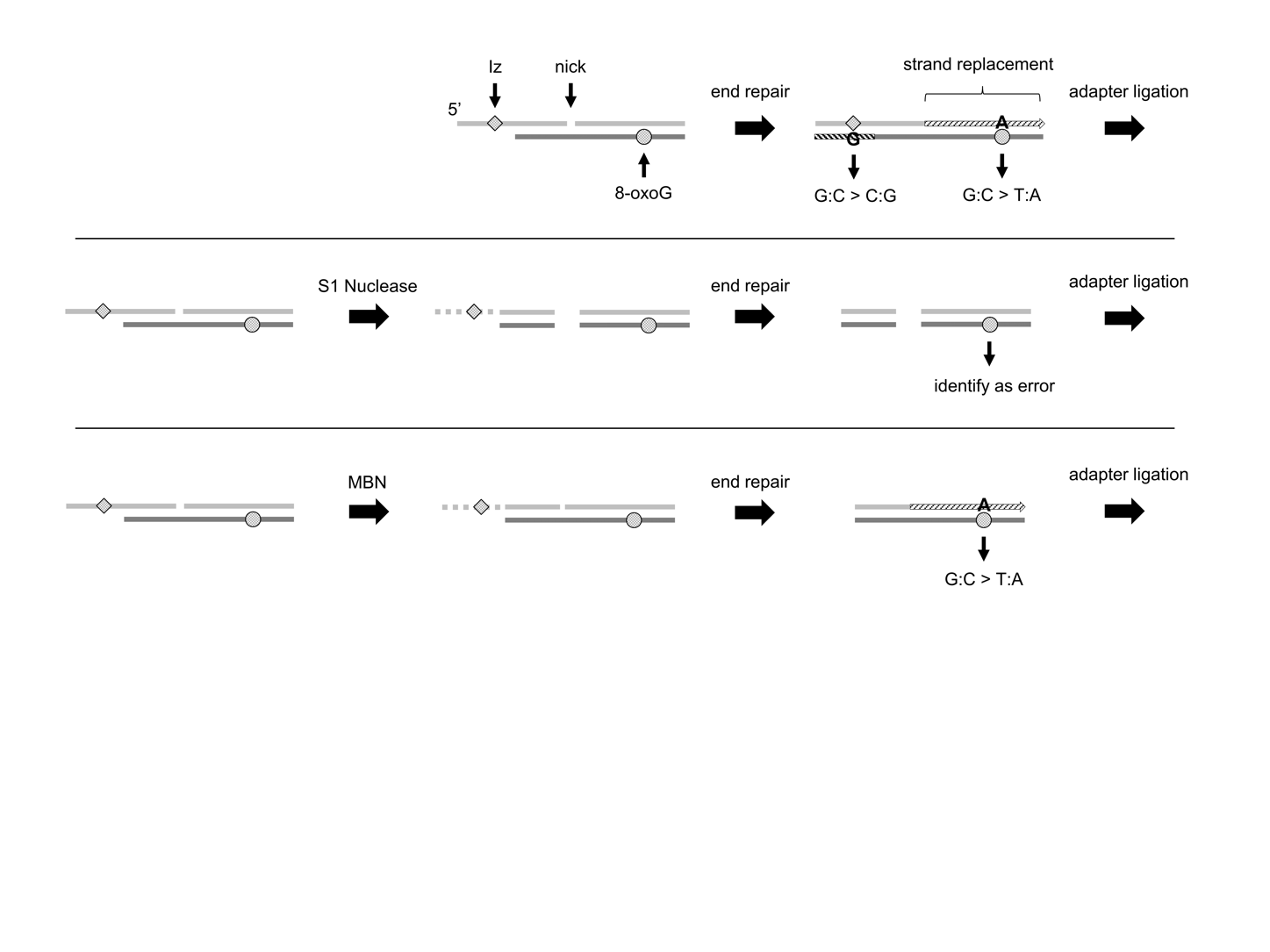


**Supplementary Fig. S4**

Hypothesis regarding differences in the G>T and G>C error reduction ability between MBN and S1 Nuclease based on substrate specificity for SS regions by S1 Nuclease and MBN. (top) DNA strand around nicked sites would be replaced in the 5ʹ->3ʹ direction during the end repair process. If the template strand has an 8-oxoG, adenine will be misincorporated into the complementary strand, which cause the G:C>T:A mutation to occur. Iz might occur frequently in naked SS regions, which induce the G:C>C:G mutation. (middle) S1 Nuclease can degrade all these SS regions effectively, thereby decrease these errors. (bottom) MBN can degrade SS DNA, but not nicked sites, thereby reduce G:C>C:G errors, but not G:C>T:A errors effectively

**References**

Matsumura S, Sato H, Otsubo Y, Tasaki J, Ikeda N, Morita O (2019) Genome-wide somatic mutation analysis via Hawk-Seq^TM^ reveals mutation profiles associated with chemical mutagens. Arch Toxicol 93:2689–2701. <https://doi.org/10.1007/s00204-019-02541-3>
